# Supplementary material for: Causal Relationship between Aging and Anorexia Nervosa: A White-Matter-Microstructure-Mediated Mendelian Randomization Analysis
Source: Biomedicines. 2024 Aug 16;12(8):1874. doi: 10.3390/biomedicines12081874 (PMC11351342; doi:10.3390/biomedicines12081874)
Supplement: Supplementary file 1 [file biomedicines-12-01874-s001.zip › Supplementary Figure/Figure S9/Leaveoneout plot of MR analysis for ubm-b-1958 to AN.pdf]

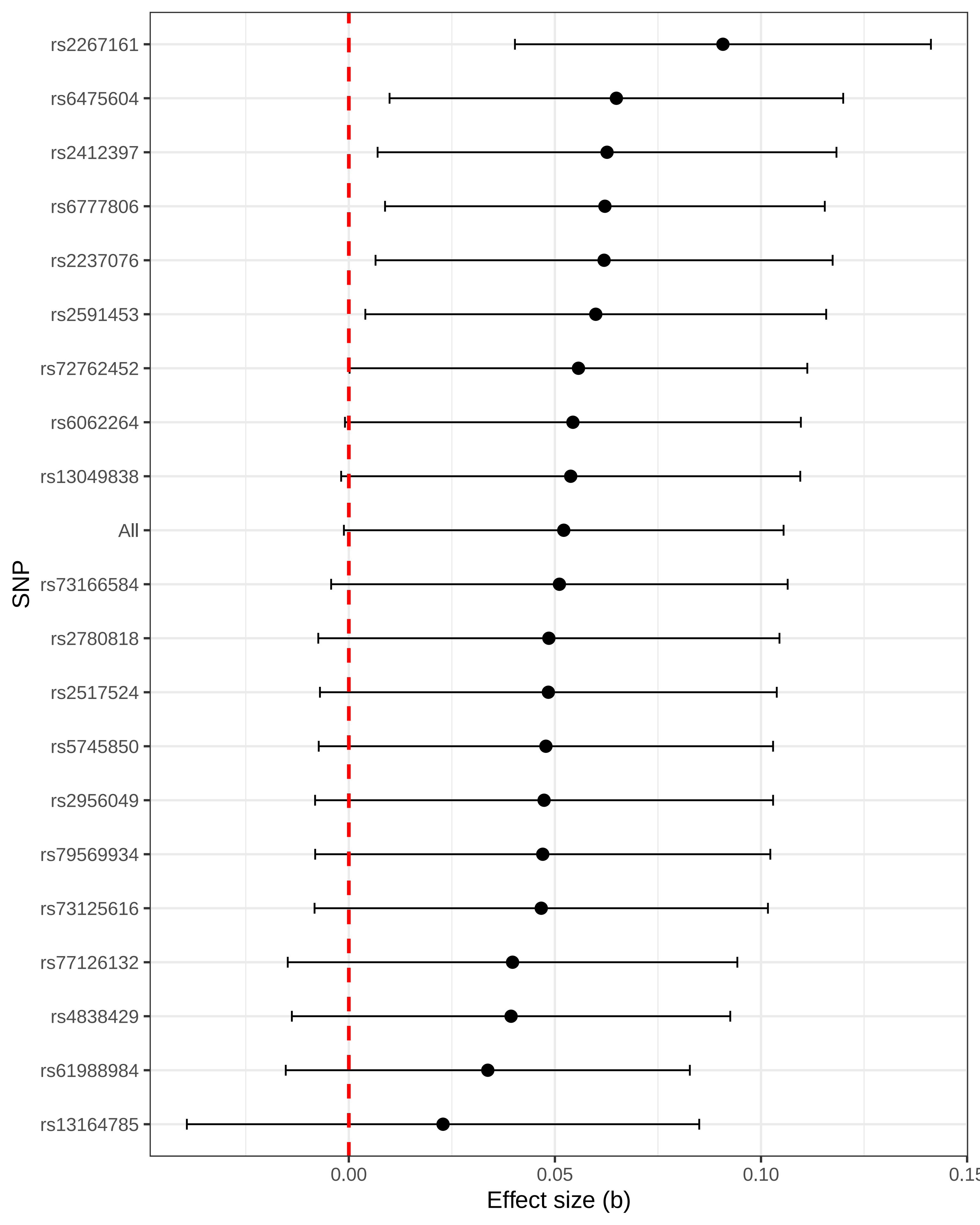
SNP
rs2267161
rs6475604
rs2412397
rs6777806
rs2237076
rs2591453
rs72762452
rs6062264
rs13049838
All
rs73166584
rs2780818
rs2517524
rs5745850
rs2956049
rs79569934
rs73125616
rs77126132
rs4838429
rs61988984
rs13164785
0.00
0.05
0.10
0.15
Effect size (b)
